# Design of Embedded-Hybrid Antimicrobial Peptides with Enhanced Cell Selectivity and Anti-Biofilm Activity

**DOI:** 10.1371/journal.pone.0098935

**Published:** 2014-06-19

**Authors:** Wei Xu, Xin Zhu, Tingting Tan, Weizhong Li, Anshan Shan

**Affiliations:** Laboratory of Molecular Nutrition and Immunity, Institute of Animal Nutrition, Northeast Agricultural University, Harbin, Heilongjiang, China; University of Cambridge, United Kingdom

## Abstract

Antimicrobial peptides have attracted considerable attention because of their broad-spectrum antimicrobial activity and their low prognostic to induce antibiotic resistance which is the most common source of failure in bacterial infection treatment along with biofilms. The method to design hybrid peptide integrating different functional domains of peptides has many advantages. In this study, we designed an embedded-hybrid peptide R-FV-I16 by replacing a functional defective sequence RR7 with the anti-biofilm sequence FV7 embedded in the middle position of peptide RI16. The results demonstrated that the synthetic hybrid the peptide R-FV-I16 had potent antimicrobial activity over a wide range of Gram-negative and Gram-positive bacteria, as well as anti-biofilm activity. More importantly, R-FV-I16 showed lower hemolytic activity and cytotoxicity. Fluorescent assays demonstrated that R-FV-I16 depolarized the outer and the inner bacterial membranes, while scanning electron microscopy and transmission electron microscopy further indicated that this peptide killed bacterial cells by disrupting the cell membrane, thereby damaging membrane integrity. Results from SEM also provided evidence that R-FV-I16 inherited anti-biofilm activity from the functional peptide sequence FV7. Embedded-hybrid peptides could provide a new pattern for combining different functional domains and showing an effective avenue to screen for novel antimicrobial agents.

## Introduction

Antimicrobial peptides (AMPs) are a series of small proteins (10-40 amino acids residues) that could be used as potent weapons against infectious microbes, including multidrug-resistant bacteria [1]. As novel biomaterials, peptides provide excellent avenues for a wide range of bioengineering and biomedical applications [2], [3], including regenerative biomaterials, therapeutic delivery, and antimicrobial agents [4]–[7]. Thousands of AMPs with bactericidal property have been isolated from a wide range of species. Despite huge variations in length and primary structure, natural AMPs exhibit common features such as net positive charges (typically ranging from +2 to +9) and amphipathicity [8], [9]. However, the membrane-active mechanism of the cationic peptides has not been fully understood, and the cost of synthesis and limited function compromise their development as antimicrobial agents.

Bacteria growing on surfaces form biofilms, a complex bacterial lifestyle adaptation that provides a protection from environmental stresses [10]–[13]. Accompanying with biofilms formation, bacterial infections are extremely difficult to treat, because of the general antibiotic resistance of subpopulations in the biofilm community, which are regarded as antibiotic-resistant “superbugs” [14]. A breakthrough observation showed that the natural human cathelicidin peptide LL-37 had the capability to block *Pseudomonas aeruginosa* biofilm growth and accelerate the disintegration of preformed biofilms [15]. As previously reported, several peptides integrating sequence FV7 (FRIR VRV) have been tested positively for anti-biofilm activity. Therefore, the sequence FV7 can serve as a basis for the iterative design of improved peptides [16].

Amphipathicity is recognized as a prerequisite for α-helical AMPs activity, however, perfect amphipathicity possibly results in a simultaneous increase in the bactericidal activity and cytotoxicity [17]. Recently, it has been showed that disrupting perfect amphipathicity of α-helical structure can maintain strong antimicrobial activity, reduce hemolytic activity and promote pore formation [18]–[20]. In general, various approaches have been employed to optimize native AMPs, e.g., as a novel design, hybrid peptides could combine the advantages of different peptides [21]. With inherent amphiphilic property and the capability to aggregate in bulk solution, hybrid peptides show great potential at interfaces [22]. However, there are few patterns to describe hybrid peptides, disruption of amphipathic structure and anti-biofilm activity.

**Figure 1:**
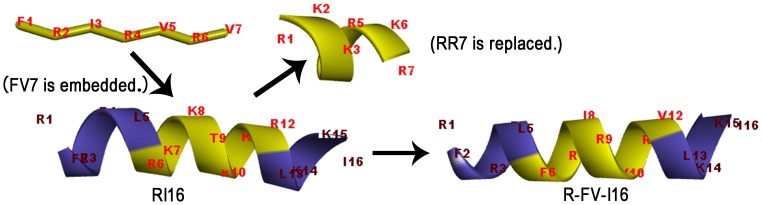
Three-dimensional structure projections of peptides and the process of embedded-hybrid.

Porcine myeloid antibacterial peptide, PMAP-36(1–20), as one of well-known cationic peptides, displayed antibacterial activity against bacteria *in vitro* [23], [24]. The peptide RI16 (RFRR LRKK TRKR LKKI) is truncated from PAMP-36(1–20) which presented an inherently perfect amphipathic structure, and previous experiments demonstrated that RI16 retained antibacterial activity similar to PMA-36(1–20) [20]. Generally, hybrid peptides serially combined different functional sequences, and this resulted in increasing the length of peptides. In this study, we applied a novel pattern that embedded-hybrid peptides with imperfect amphipathicity were designed by replacing the original sequence and embedding FV7 in the central position of RI16, as shown in Table 1 and Fig. 1. We hypothesized that this hybrid peptide could enhance antibacterial activity and inherit the anti-biofilm activity from FV7. The C-terminus of all the peptides was aminated to improve the stability and eliminate potential electrostatic attractions [25]. We first measured the secondary structure of peptides in solution (phosphate buffer) and membrane-mimicking environments (sodium dodecyl sulfate and trifluoroethyl alcohol, SDS and TFE, respectively). The antibacterial activity was determined against broad model microbes, including Gram-negative bacteria (*Escherichia coli, Salmonella typhimurium, Salmonella pullorum* and *P. aeruginosa*) and Gram-positive bacteria (*Staphylococcus aureus, Staphylococcus epidermidis*, *Streptococcus faecalis* and *Bacillus subtilis*). The anti-biofilm activity against *P. aeruginosa* and *E*. *coli*, the hemolytic effect on human red blood cells (hRBCs), the cytotoxicity assay and the salt sensitivity and serum stability were then measured. A series of assays about the inner/out membrane and the cytoplasmic membrane were performed to investigate the potential peptide-membrane interaction mechanisms, and scanning electron microscopy (SEM) and transmission electron microscopy (TEM) could visualize those results. As a well-known peptide, the bee venom melittin (ME26) has a strong antibacterial activity and kills bacterial strains via a toroidal-pore mechanism [26], served as a control peptide in this study for studying the membrane-active mechanism. The results indicated that the hybrid peptide R-FV-I16 with the embedded anti-biofilm sequence FV7 increased the cell selectivity and retained the anti-biofilm activity.

**Table 1:** Peptide design and their key physicochemical parameters.

Peptides	Sequence	TTheoretical MW	Measured MW^a^	Net charge
RI16	RFRR **LRKK TRKR** LKKI-NH_2_	2182.77	2224.84	+12
R-FV-I16	RFRR **LFRI RVRV** LKKI-NH_2_	2156.74	2197.82	+8
FV7	**FRIR VRV**-NH_2_	945.18	986.24	+4
RR7	**RKKT RKR**-NH_2_	972.19	1013.27	+5

a Molecular weight (MW) was measured by mass spectroscopy (MS).

## Materials and Methods

### Peptide synthesis

All the peptides used in this study were synthesized and purified by GL Biochem (Shanghai, China) using N-9-Fluorenylmethyloxycarbonyl (Fmoc) chemistry. The purity was measured to be greater than 95% using analytical reverse-phase high-performance liquid chromatography (RP-HPLC). The molecular masses of peptides were confirmed using matrix-assisted laser desorption/ionization time-of-flight mass spectroscopy (MALDI-TOF MS, Linear Scientific Inc., U.S.A.). Peptides were dissolved in deionized water (DI water) at a concentration of 2.56 mM and stored at −20°C.

### Peptide design and sequence analysis

**Figure 2:**
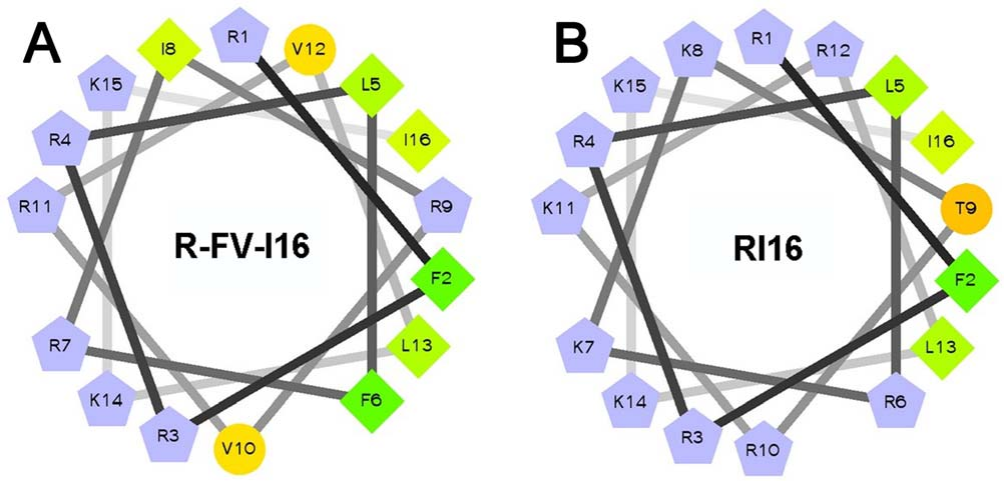
Helical wheel projections of the peptides. By default the output presents the hydrophilic residues as circles, hydrophobic residues as diamonds, potentially negatively charged as triangle, and potentially positively charged as pentagons. Hydrophobicity is color coded as well: the most hydrophobic residue is green, and the amount of green is decreasing proportionally to the hydrophobicity, with zero hydrophobicity coded as yellow. Hydrophilic residues are coded red with pure red being the most hydrophilic (uncharged) residue, and the amount of red decreasing proportionally to the hydrophilicity. The potentially charged residues are light blue.

First, RI16 was obtained through being truncated from α-helical peptide PMAP-36(1–20), which is a 16-residue perfectly amphipathic parental peptide. Secondly, an anti-biofilm sequence FV7 was embedded in the peptide RI16 to substitute RR7 (RKKT RKR). Aimed sequence RR7 locates at the center of the primary structure, meanwhile this location is also in the polar face of the amphipathic α-helical peptide RI16. As demonstrated by previous studies, the middle position of the α-helical AMPs is strategic and important in designing and/or optimizing AMPs [20], [27]. The amphipathic sequence FV7 disrupted the amphipathicity of RI16, as shown in Fig. 2.

The primary structure analysis of the peptides was performed online using EMBOSS Pepinfo (http://www.ebi.ac.uk/Tools/seqstats/emboss_pepinfo/). The mean hydrophobicity and relative hydrophobic moment were calculated online using CCS scale (http://www.bbcm.univ.trieste.it/~tossi/HydroCalc/HydroMCalc.html). The secondary structure type for each residue was predicted online using Jpred 3 (http://www.compbio.dundee.ac.uk/www-jpred/index.html). The helical wheel projection was performed online using the Helical Wheel Projections: http://rzlab.ucr.edu/scripts/wheel/wheel.cgi.

### Circular dichroism (CD) measurements

The solutions were prepared at a 150 µM peptide concentration in 10 mM PBS (pH 7.4, to mimic an aqueous environment), 50% TFE (to mimicthe hydrophobic environment of the microbial membrane) (Sigma), and 30 mM SDS micelles (negatively charged prokaryotic membrane comparable environment) (Sigma). The CD spectra were measured with a CD spectropolarimeter (Jasco, J-820, Tokyo, Japan) at 25°C, using a 0.1 cm path length rectangular quartz cell. The spectra were recorded at between 190 and 250 nm at 10 nm/min scanning speed. At least three scans were conducted for each peptide sample.

The acquired CD spectra were converted to the mean residue ellipticity through the following equation: 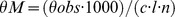



where θM is the mean residue ellipticity [deg.cm2.dmol-1], θobs is the observed ellipticity corrected for the buffer at a given wavelength [mdeg], c is the peptide concentration [mM], l is the path length [mm], and n is the number of amino acids.

The percentage of α-helical structure was calculated as followings: 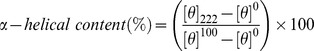



where [θ]_222_ is the experimentally observed mean residue ellipticity at 222 nm, and values for [θ]^0^ and [θ]^100^ which correspond to 0 and 100% a-helix content at 222 nm, are estimated to be −2000 and −32000, respectively [28], [29].

### Antimicrobial assays

Minimum inhibitory concentration (MIC), as the minimal peptide concentration that completely inhibited bacterial growth, was measured by using a modified version of the Clinical Laboratory and Standards Institute (CLSI) broth microdilution method as described previously [30]. *E. coli* ATCC25922, *P. aeruginosa* ATCC27853, *S. aureus* ATCC29213, *S. epidermidis* ATCC12228, *S. faecalis* ATCC29212 and *B. subtilis* CMCC63501 were obtained from the College of Veterinary Medicine, Northeast Agricultural University (Harbin, China), *E. coli* K88, *E. coli* CVCC1515 *and P. aeruginosa* ATCC15442 were bestowed by Prof. J.H. Wang (Gene Engineering Laboratory, Feed Research Institute, Chinese Academy of Agricultural Sciences Beijing, China) and *E. coli* UB1005 was kindly provided by Prof. Q.S. Qi (State Key Laboratory of Microbial Technology, Shandong University, China). In this study, antibiotic-resistant variants of *P. aeruginosa* ATCC15442 were developed by step-wise culture at 37°C in MHB with selected antibiotics (ciprofloxacin hydrochloride and gentamicin) at 2, 4, 6, 8 and at least 20 times the MIC. Resistant variants obtained were then routinely cultured in MHB containing the respective antibiotic at least 20 × MIC for 24 h at 37°C. Briefly, 50 µl of approximate 0.5–1 × 10^6^ colony forming units (CFU)/ml of cells were diluted in Mueller-Hinton broth and distributed to 96-well plate, 50 µl of twofold peptides were added and the plates was incubated at 37°C for 18–24 h. The MIC values were calculated as the lowest peptide concentration that prevented visible turbidity and negative control of the pure broth without microbes. The tests were performed at least three times in duplicate.

### Kinetics of Bacterial Killing

Kinetics of the bacterial killing was measured by using *E. coli* ATCC25922 in logarithmic phase (10^8^ CFU/mL). The above bacteria were diluted to 1/1000 with PBS, and 500 µL solutions was added with 500 µL PBS at different final concentrations (1/4, 1/2 and 1 × MIC). The solutions were incubated for 0.5 min, 1 min, 2.5 min, 5 min, 10 min, 20 min, and 40 min at 37°C. The moderately diluted samples were plated on nutrient agar plate (MHA). The bacterial colonies were counted after incubation for 18 h at 37°C. The results were determined and presented as the mean of three repeats.

### Measurement of hemolytic activity

The amount of released hemoglobin by the lysis of erythrocytes was measured for hemolytic activity of peptides. Human erythrocytes were obtained from a healthy donor with written informed consent. The experimental protocol was reviewed and approved by the ethics committee of the Northeast Agricultural University Hospital. The measurement was based on a previously described method [31]. The fresh hRBCs were washed three times by centrifugation (1000 g, 5 min, 4°C) in PBS (pH 7.4), and then resuspended in PBS to obtain the erythrocyte with a dilution of approximately 1% (v/v). Then, 50 µL of hRBCs solution was incubated with 50 µL of different concentrations of the peptides dissolved in PBS for 1 h at 37°C, before centrifugation (1000 g, 5 min, 4°C) again. The absorbance of the supernatants was measured at optical density (OD) 492 nm. The concentration causing 10% hemolysis was defined as the minimal hemolytic concentration (MHC). The control samples for 0% and 100% hemolysis consisted of hRBCs in PBS (A_blank_) only and in 0.1% Triton X-100 (A_triton_), respectively. The percentage of hemolysis was calculated according to the following equation:
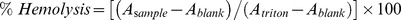



### Cytotoxicity assay

Peptides can inhibit proliferation of cell, called cytotoxicity. The colorimetric 3-(4, 5 dimethylthiazol-2-yl)-2, 5-diphenyltetrazolium bromide (MTT) (Sigma) dye reduction assay was used to determine the cytotoxicity of peptides on piglets' intestinal epithelium cells (PIEC) and human embryonic kidney 293 cells (HEK293) according to a previously described method [32]. The PIEC was kindly bestowed by Prof. Y.M. Zhang (College of Veterinary Medicine, Northwest A&F University, China) and HEK293 was kindly bestowed by Prof. N. Wang (College of Animal Science and Technology, Northeast Agricultural University, China). Briefly, the cells were seeded on a 96-well plate at a density of 2 × 10^5^ cells/ml in Dulbecco modified eagle medium (DMEM). After incubation about 8 h, 10 µl of peptides with varying concentrations (2, 4, 8, 16, 32, 64, 128, 256 µM) solution were added. And then, the cells were incubated under a fully humidified atmosphere of 95% air and 5% CO_2_ at 37°C about 22–24 h. Cell cultures were incubated with MTT (50 µl, 5 mg/ml) for 4 h at 37°C. After centrifugation (1000 g, 5 min), and the supernatants of cell cultures were discarded. Subsequently, 150 µl of dimethyl sulfoxide (DMSO) was added to dissolve the formazan crystals formed, and the OD was measured using a microplate reader (Bio-Tek Instruments Inc., USA) at 570 nm.

### Stability study

The stability of peptides was tested in the MIC assay in different environments, including salt sensitivity, human serum, thermal stability and susceptibility to enzymes. For salt sensitivity, 0.5–1 × 10^6^ CFU/ml of *P. aeruginosa* ATCC27853 and *E. coli* ATCC25922 were treated with peptides, while different salts were added to MHB, and the final concentrations of physiological salts were as follows: 150 mM NaCl, 4.5 mM KCl, 1 mM MgCl_2_, 6 µM NH_4_Cl, 8 µM ZnCl_2_, 4 µM FeCl_3_ and 2.5 µM CaCl_2_ [33]. Except salt sensitivity, only *E. coli* ATCC25922 was treated with peptides. For testing MIC of peptides in serum environment, human serum was dissolved in the MHB to reach the final concentrations (25%, 50%). For testing peptide sensitivity to the proteolytic enzymes, the peptides were incubated in advance at 37°C for 1 h with a 1 mg/ml final concentration of the proteolytic enzymes (trypsin, pepsin, papain and proteinase K). For testing thermal stability, incubation occurred at 100°C for 1 h. After these treatments, the procedures were same as MIC assay described above.

### Evaluation of the outer membrane permeability

The outer membrane (OM) of Gram-negative bacteria provides important protection for the organism. We evaluated the change of OM permeability induced by peptides, using N-phenyl-1-naphthylamine (NPN), a membrane potential-sensitive dye, as previously described [34]. Briefly, *E. coli* UB1005 cells grown to logarithmic phase in MHB, suspended in 5 mM of sodium HEPES buffer (pH 7.4, containing 5 mM of glucose), and diluted to 10^5^ CFU/ml in the same buffer. NPN was added to bacteria at a final concentration of 10 µM. The background fluorescence was recorded (excitation λ = 350 nm, emission λ = 420 nm). The changes were recorded by using an F-4500 fluorescence spectrophotometer (Hitachi, Japan). Peptides were added to the quartz cuvette to give a different final concentration (0.5–8 µM). The fluorescence was recorded as a function of time until no further increase.

### Evaluation of the inner membrane permeability

As previously described, ONPG can penetrate into cytoplasm where it is cleaved to o-nitrophenol (ONP) by cytoplasmic β-galactosidase. Then ONP is released into the culture medium, and its intensity reflected the permeabilization of the inner membrane. *E. coli* UB1005 was cultured at 37°C to the logarithmic phase in MHB medium containing 2% lactose and harvested by centrifugation (20001 g, 10 min). The cells were washed twice, re-suspended in the buffer (10 mM of PBS with pH 7.4, 1.5 mM of ONPG) and diluted to an OD_600_ of 0.05. The cells were incubated with different concentrations (0, 1/2 and 1 × MIC). OD value was measured at 420 nm from 0 to 30 min every two minutes, reflecting the ONPG flowed into the cells as a permeability indicator of the inner membrane [20].

### Cytoplasmic membrane electrical potential measurement

Peptides could alter the electrical potential of cytoplasmic membrane, and this potential that was measured by using *E. coli* UB1005 cells and diSC_3_-5 (Sigma), a membrane potential-sensitive fluorescent dye, as previously described [20], [35]. *E. coli* UB1005 was cultured to logarithmic phase in MHB, harvested by centrifugation, washed twice with buffer (5 mM of HEPES and 20 mM of glucose, pH 7.4) and resuspended to an OD_600_ of 0.05 in the identical buffer. The dye (diSC_3_-5) was added to above buffer at a final concentration of 0.4 µM. And the bacteria were incubated to take up the dye for 90 min. KCl was added to a final concentration of 0.1 M for equilibrating K^+^ concentration between extracellular and intracellular environment, and then the bacteria were incubated at room temperature for 10 min. The cell suspension (2 ml) was placed in a 1-cm cuvette, and the peptides were added to different concentrations. The fluorescence intensity changes were monitored by using an F-4500 fluorescence spectrophotometer (Hitachi, Japan) with an excitation wavelength of 622 nm and an emission wavelength of 670 nm.

### Scanning electron microscopy (SEM) observations

For the SEM sample preparation, the bacterial cells of *E. coli* ATCC25922 were cultured to a logarithmic phase in MHB at 37°C under constant shaking at 220 rpm, harvested by centrifugation (1000 g, 10 min), washed twice with 10 mM PBS, and resuspended to an OD_600_ of 0.2. The cells were incubated at 37°C for 30 and 120 min with R-FV-I16 at 1 × MIC, respectively. The control was run without peptides. After incubation, the cells were centrifuged at 5000 g for 5 min. The cell pellets were then washed three times with PBS, and centrifuged similarly after each wash. Following that, the cell pellets were subjected to fixation with 2.5% glutaraldehyde at 4°C, about 6 to 8 hours, followed by washing with PBS twice. Then, the cells were dehydrated for 10 min in a graded ethanol series (50, 70, 90, and 100%) and for 15 min in 100% ethanol, a mixture (1∶1) of 100% ethanol and tertiary butanol, and absolute tertiary butanol. Then the specimens were dehydrated, dried, coated with gold, and examined using a HITACHI S-4800 FE-SEM.

### Transmission electron microscope (TEM) observations

The preparation of bacteria samples was conducted in the same manner as for the SEM observations. After pre-fixation with 2.5% glutaraldehyde at 4°C overnight, the specimens were post-fixed with 2% osmium tetroxide for 45 min. Both fixations were followed by washing with PBS twice (15 min each). Then, the bacteria samples were dehydrated using a series of graded ethanol solutions as described above. The specimens were then transferred to 1∶1 mixtures (absolute acetone and epoxy resin) for 30 min and incubated overnight in pure epoxy resin at the certain temperature. The specimens were sectioned with ultramicrotome, stained by uranyl acetate and lead citrate and examined by using a HITACHI H-7650 TEM.

### Effect of peptides on biofilm formation and image observations

As described previously, a static biotic solid surface assay shows the capability of peptides to inhibit biofilm formation [15], [36]. *P. aeruginosa* ATCC27853 and *E. coli* ATCC25922 bacteria were cultured in Tryptic Soy Broth (TSB) overnight and dilutions (1/100) of the colony were distributed into 96-well plate with different concentrations of peptides, incubated for 22 h at 37°C. The medium with planktonic cells was discarded and the wells were rinsed twice with PBS (pH 7.4). The biofilm cells adhering to the side of tubes were stained with crystal violet for 20 min and the wells were rinsed repeatedly with DI water until the water was clear without visible purple color. The stained crystal violet was dissolved in ethyl alcohol, and then measured at OD 570 nm (OD_570_), using a microplate reader (Bio-Tek Instruments Inc., USA).

For image observation, the medium with *P. aeruginosa* ATCC27853 was added to a12-well plate with a climbing film/circular cover-slip in the bottom and then each well was added with different concentrations of peptides, and dilutions (1/100) of the overnight colony were distributed into every well. After incubation for 22 h, the medium with planktonic cells was discarded and the residual biofilm was rinsed with PBS, fixed with 2.5% glutaraldehyde and subjected to dehydration using a series of graded ethanol solutions as described above. The specimens were dehydrated, dried, coated with gold and examined using a HITACHI S-3400 SEM.

### Viability assays of the preformed biofilm cells treated with peptides

The low concentration of peptides could inhibit the growth of biofilms, and the high dose is necessary to kill preformed biofilm cells. As described previously, viability assays were used to measure the effect of the peptide R-FV-I16 on preformed biofilm cells of *P. aeruginosa* ATCC27853 and *E. coli* ATCC25922 [37]. Briefly, a 12-well plate was filled with 600 µl of 10^6^ CFU/ml in TSB and incubated at 37°C for 12 h. The medium was discarded and the wells were rinsed twice with PBS (pH 7. 4). Peptide-dissolved TSB was added (600 µl). After incubation at 37°C for 12 h, the supernatant was discarded and 200 µl of PBS was added with 50 µg MTT. Viability assay is based on the formation of insoluble purple formazan from reduction of MTT by respiratory reductases of living staphylococcal cells, and the formazan crystals were dissolved in DMSO. The absorbance was measured at OD_570_ with a microplate reader (Bio-Tek Instruments Inc., USA).

## Results

### Peptide design and characterization

The cationic host defense peptide, RI16 has a perfect amphipathic helical structure. FV7, with 3 cationic residues and 4 hydrophobic amino acids, is analogous to cationic host defense peptides. In this study, we selected two cationic peptides as parents (RI16 and FV7) for designing hybrid peptide. The wheel projections from Fig. 2 show that cationic residues of RI16 centralized on one side of the helix, whereas the hydrophobic amino acid residues are on the other side. Therefore, RI16 presents a perfect amphipathic structure. RI16 showed poor antimicrobial activity and anti-biofilm activity. However, after we substituted FV7 for RR7, the perfect amphipathic nature of the α-helical structure was disrupted. We speculated that this new structure could enhance cell selectivity and retain anti-biofilm activity. This way could also make hybrid peptide retain identical length. Therefore, cationic embedded-hybrid peptide R-FV-I16 was designed in the following way: a functional sequence FV7 was embedded into RI16 and replaced the defective sequence RR7. The molecular weight (MW) of the peptide was verified by MALDI-TOF MS, as shown in Table 1.

### Secondary structure

**Figure 3:**
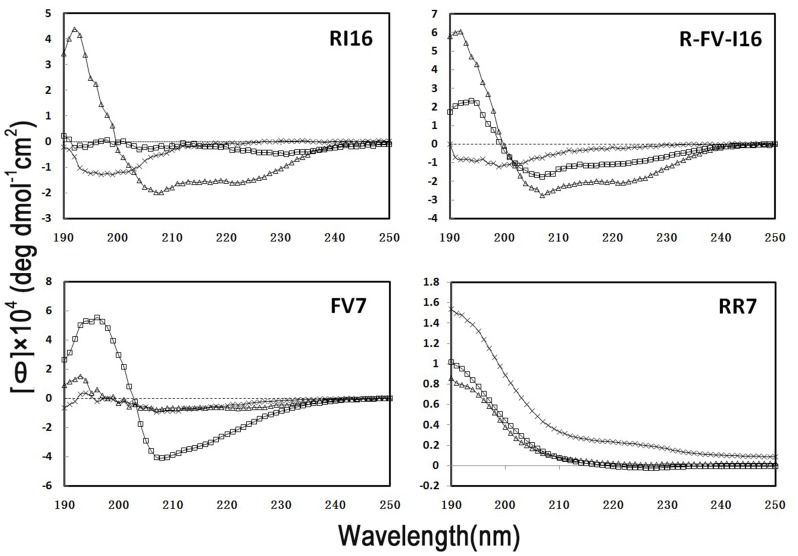
The CD spectra of the peptides. The peptides are dissolved in 10(pH 7.4) (×, dash lines), 50% TFE (△, triangles), or 30 mM SDS (□, squares), and the concentrations are fixed at 150 µM. Data are expressed as the mean residue ellipticities.

It has been reported that PMAP-36 forms an α-helical structure in membrane-mimetic environments such as TFE, SDS micelles or liposome [38]. The secondary structure of the peptides in different environments was investigated by CD spectroscopy. Fig. 3 showed the CD spectra of the peptides at 150 µM of PBS (pH 7.4, 10 mM), 50% TFE, and SDS (30 mM). According to the results, all of peptides retained a random coil structure in sodium phosphate buffer. In TFE, R-FV-I16 and RI16 displayed one positive (approximately 195 nm) and two negative (approximately 208 and 220 nm) bands, which typically represented α-helix structures. R-FV-I16 and RI16 contained the different α-helical content, 69% and 53%, respectively. R-FV-I16 also displayed α-helix structures (36%) in the SDS environment. However, the structure of parental peptide RI16 could not be observed in the SDS environment. The CD spectra of parental peptide FV7 significantly demonstrated a β-sheet in the SDS micelles, with a maximum near 200 nm and a minimum just below 220 nm [39].

### Antimicrobial activity and hemolytic activity

The antimicrobial activity of different peptides against Gram-negative and Gram-positive strains is summarized in Table 2. AMP R-FV-I16 exhibited the potent antimicrobial activity against all the bacterial strains in the assays, with MICs ranging from 1 to 4 µM. Peptide FV7 exhibited the moderate capability of killing bacterial strains with MICs ranging from 8 to 64 µM. Peptides PMAP-36(1-20), RI16, and RR7 showed poor antimicrobial activity. Moreover, results showed that selected antibiotics (ciprofloxacin hydrochloride and gentamicin) showed poor antimicrobial activity to resistant variants of *P. aeruginosa*, with MICs above 256 µM. The calculated geometric mean (GM) obtained by MICs for all tested strains in the experiment, reflects the therapeutic effect of the peptide to typical bacterial strains in clinic. The GM values of RI16 and FV7 were about 60 and 15 times higher than that of R-FV-I16, respectively. It demonstrated that R-FV-I16 had a more remarkable improvement than parental peptides RI16 and FV7, in terms of antimicrobial activity. The hemolytic activity of the peptides was determined at varying concentrations. The results are shown in Table 2.

**Table 2:** Antimicrobial and hemolytic values of the peptides.

	RI16	R-FV-I16	FV7	RR7	ME26
***MIC*** a **(** ***µM*** **) Gram-negative bacteria**					
*E. coli* ATCC25922	128	1	16	>256	2
*E. coli* K88	>256	2	16	>256	2
*E. coli* UB1005	128	4	32	>256	2
*E. coli* CVCC1515	128	2	32	>256	2
*P. aeruginosa* ATCC27853	128	4	16	>256	2
*P. aeruginosa* ATCC15442	>256	2	16	>256	2
*P. aeruginosa* variant 1^b^	>256	2	8	>256	4
*P. aeruginosa* variant 2^c^	>256	4	16	>256	8
*S. typhimurium* ATCC14028	64	2	16	>256	4
*S. pullorum* C79-13	64	1	128	>256	2
Gram-positive bacteria					
*S.aureus* ATCC29213	128	2	64	>256	8
*S.epidermidis* ATCC12228	32	4	32	>256	0.5
*S.faecalis* ATCC29212	128	1	32	>256	1
*B. subtilis* CMCC63501	64	1	16	>256	1
***MHC*** d ***(µM)***	>256	128	>256	>256	0.25
***GM*** e	122	2	29	256	2.2
***Therapeutic index*** f	*4.2*	*64*	*17.7*	*2*	*0.1*

a Minimum inhibitory concentrations (MIC) were determined as the lowest concentration of the peptides that inhibited bacteria growth. The tests were performed at least three times in duplicate.

b *P. aeruginosa* variant 1 was obtained by step-wise culture with ciprofloxacin hydrochloride.

c *P. aeruginosa* variant 2 was obtained by step-wise culture with gentamicin.

d MHC is the minimum concentration that caused 5% hemolysis of human red blood cells (hRBC). When no 5% hemolysis was observed at 256 µM, a value of 512 µM was used to calculate the therapeutic index.

e GM denotes the geometric mean of MIC values from all microbial strains in this table.

f Therapeutic index (TI) is the ratio of the MHC to the geometric mean of MICs (GM). Larger values indicate greater cell selectivity.

The therapeutic index (TI) is calculated by the ratio of MHC (the concentration that causes 10% hemolysis) to MIC. Larger values of the TI indicate greater cell selectivity, as shown in Table 2.

**Figure 4:**
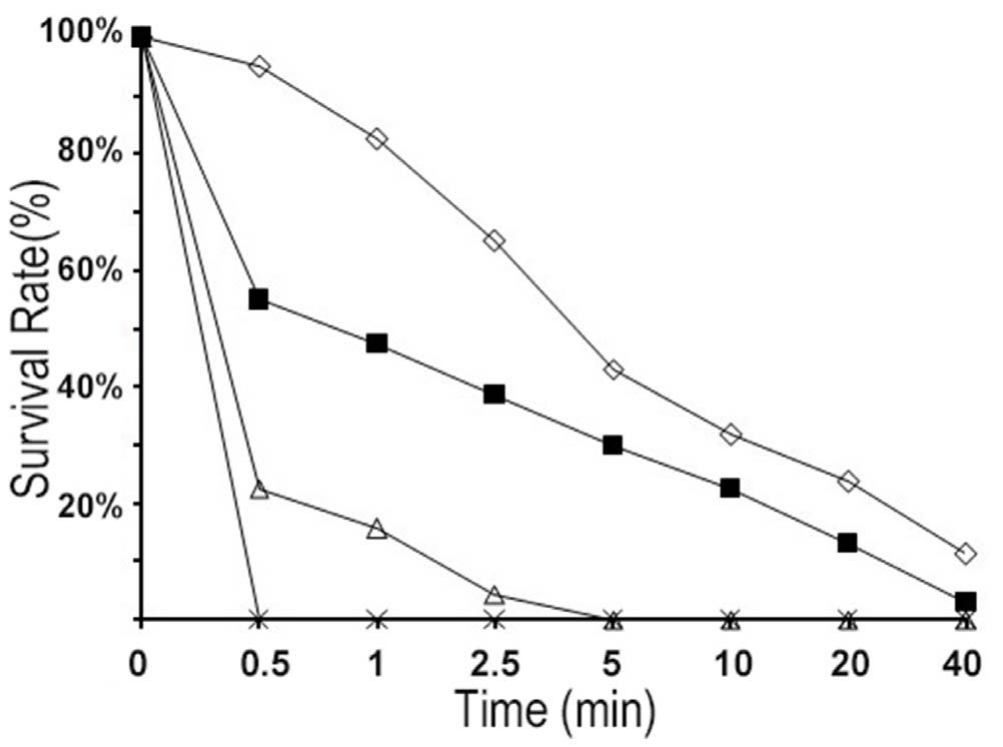
Kill kinetics of R-FV-I16 against *E. coli* ATCC 25922. The logarithmic bacteria (10^5^ CFU/ml) were separately exposed to each peptide at different final concentrations (1/4 × MIC; 1/2 × MIC; 1 × MIC) for 0.5, 2.5, 5, 10, 20, and 40 min, respectively, at 37°C in 10 mM sodium phosphate buffer (pH 7.4). The bacterial colonies were plated on nutrient agar plates. The CFU were counted after an incubation of 18 h at 37°C. Symbols: ×,2 × MIC; △,1× MIC; ▪,1/2× MIC; □,1/4× MIC.

As a function of time, the kinetics of bacterial killing are shown in Fig. 4. Within 30 min, R-FV-I16 at a final concentration (1× MIC) reduced the colony count of *E. coli* ATCC25922 by 99%. At lower concentrations (1/2 and 1/4 × MIC), the killing-rates decreased sharply.

### Cytotoxicity assay

**Figure 5:**
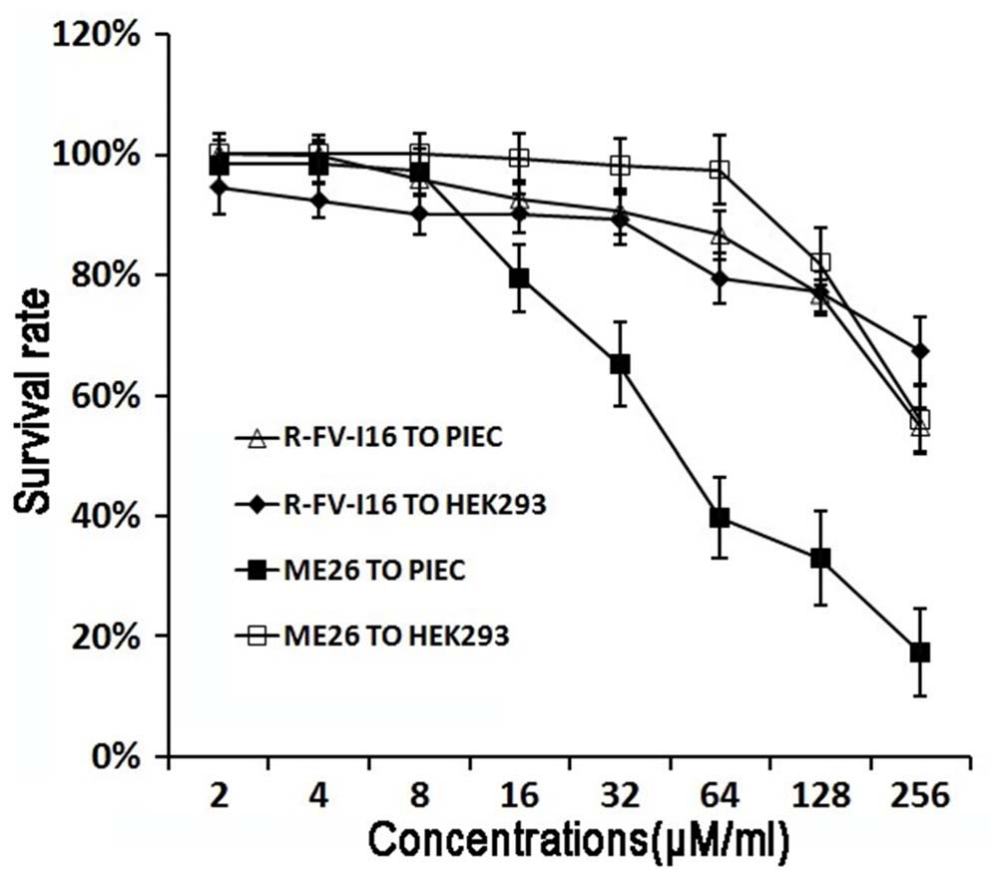
Cytotoxicity of the peptides R-FV-I16 and ME26. PIEC and HEK293 were used to evaluate the toxicity of the peptides to mammalian cells. The tests were performed at least three times.

The effect of the peptides on proliferation of PIEC and HEK293 was tested by cytotoxicity assay and results are shown in Fig. 5. The cytotoxic activity of the peptides was determined by the colorimetric MTT viability assay. The results revealed that R-FV-I16 displayed significantly lower cytotoxic activity on both cells than ME26. At the highest concentration (256 µM), R-FV-I16 displayed a reasonable cytotoxicity, with its survival rates were 67% and 55%, respecitively.

### Stability study

The antimicrobial activity of the peptides treated with different salts (physiological concentrations), serum, heat and enzymes, was tested in a stability study (Table 3 and Table 4). Salts could compromise the MIC values of peptides, especially Na^+^ and Mg^2+^. This influence was similar to both tested bacterial strains (*P. aeruginosa* and *E. coli*). Human serum, as a mixture of all kinds of salts, had a dramatically compromised effect on antimicrobial activity, especially at 50% concentration of serum. And proteolytic enzymes (trypsin, pepsin, papain and proteinase K) had a similar effect. Peptide R-FV-I16 exhibited a strong thermal stability, and the antimicrobial activity against *E. coli* remained stable after incubation at 100°C for approximately 1 h.

**Table 3:** MIC values of peptides in the presence of physiological salts^a^ with *E. coli* ATCC25922 and *P*. *aeruginosa* ATCC27853.

Peptides	Control^b^	NaCl^b^	KCl^b^	NH_4_Cl^b^	MgCl_2_ b	ZnCl_2_ b	FeCl_3_ b	CaCl_2_ b	Mix^c^
*E. coli* ATCC25922									
R-FV-I16	1	4	1	1	4	1	1	1	8
ME26	2	4	2	2	4	4	2	2	4
*P*. *aeruginosa* ATCC27853									
R-FV-I16	4	4	4	4	8	4	4	4	16
ME26	2	2	2	2	8	2	2	2	16

a Minimum inhibitory concentrations (MICs) were determined as the lowest concentration of the peptides that anti-bacteria growth.

b The final concentrations of NaCl, KCl, NH_4_Cl, MgCl_2_, CaCl_2_, ZnCl_2_, and FeCl_3_ were 150 mM, 4.5 mM, 6 µM, 1 mM, 8 µM, and 4 µM, respectively, and the control MIC values were determined in the absence of these physiological salts.

c The medium contained all kinds of salts in physiological concentrations.

**Table 4:** MIC values of peptides R-FV-I16 and ME26 treated with serum, enzymes and heat.

Peptides	Without Treatment	Enzymes				Heat	Serum	
		Trypsin	Pepsin	Caroid	Proteinase K	100°C	25%	50%
R-FV-I16	1 2	4	8	8	>128	1	4	16
ME26		>128	>128	>128	32	2	8	8

### Membrane permeability assay and electrical potential

**Figure 6:**
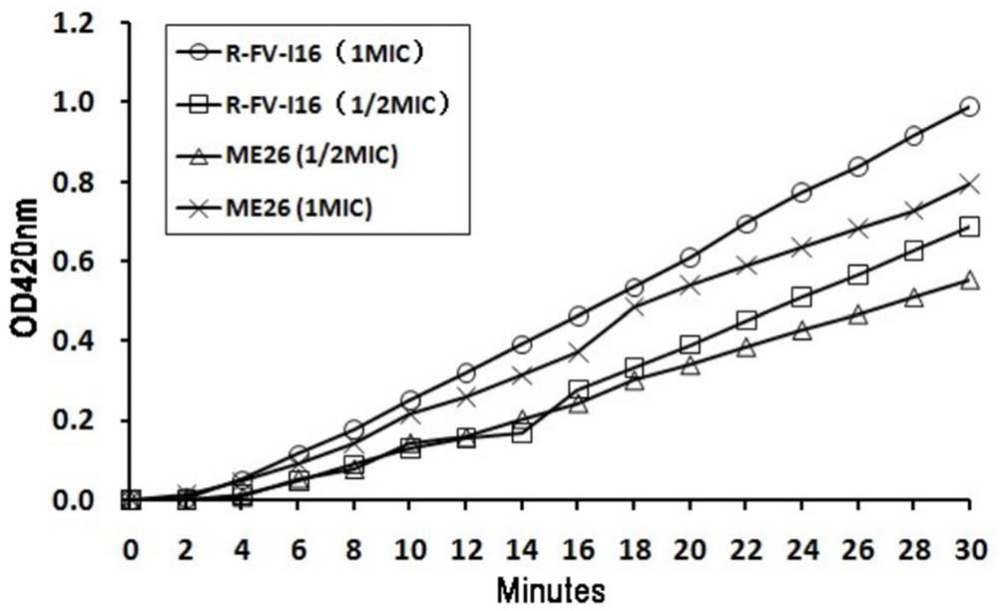
The inner membrane permeability of the peptides. ONPG was cleaved by cytoplasmic β-galactosidase of *E. coli* UB1005 treated by peptides R-FV-I16 and ME26 at 1 and 1/2 × MIC, and hydrolysate (ONP) was measured spectroscopically at absorbance of 420 nm as a function of time.

Interactions between the membrane of and AMPs are fundamental to the membrane-active mechanism of peptides [40]. Cationic AMPs could bind preferentially to the negatively charged phospholipid bilayer of bacterial cells [41], [42]. Whereas peptides induced the permeabilization of the inner membrane, ONPG entered the cytoplasm and was degraded by β-galactosidase, producing ONP that produced absorbance at 420 nm. As shown in Fig. 6, R-FV-I16 and ME26 could induce a rapid increase in the permeability of the inner membrane at their 1 × MIC, and both maintained a consistent increasing trend. The peptides R-FV-I16 and ME26 presented a considerably compromised increase at 1/2 × MIC. Compared with ME26, R-FV-I16 had an obvious effect on inner membrane permeability.

**Figure 7:**
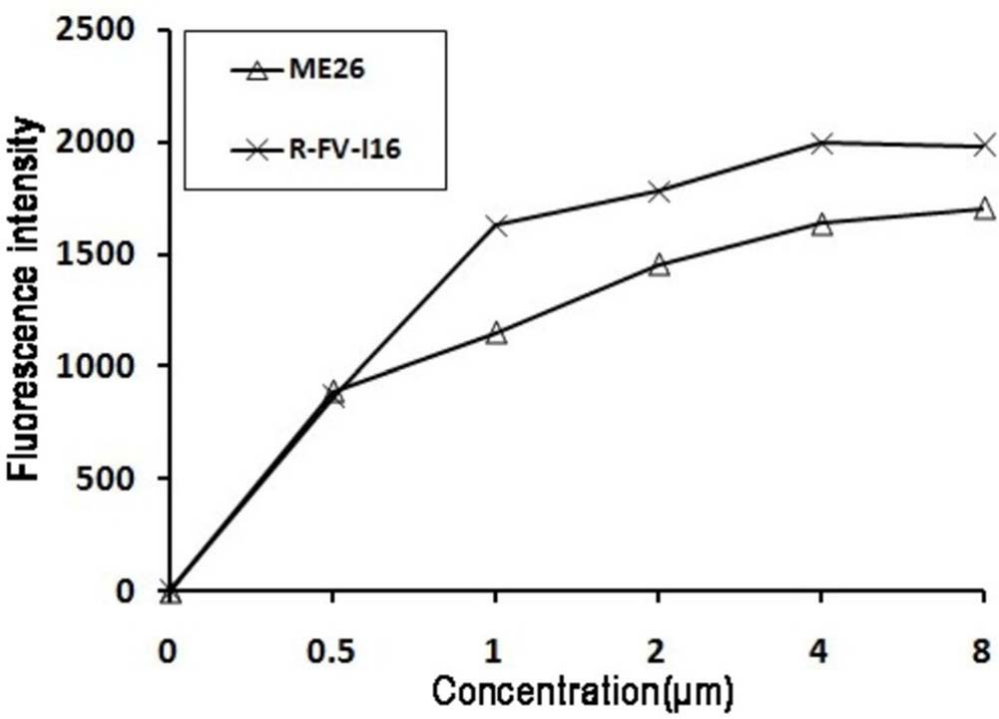
The outer membrane permeability of the peptides. The uptake of NPN of *E. coli* UB1005 in the presence of different concentrations of the peptides was determined using the fluorescent dye (NPN) assay. The NPN uptake was monitored at an excitation wavelength of 350 nm and an emission wavelength of 420 nm.

As an extra layer, the outer membrane of Gram-negative bacteria plays a crucial role in protecting the organism. Peptides could induce the permeabilization of the outer membrane and this process was evaluated by the fluorescence-based NPN uptake. The increased fluorescence was measured because dye (NPN) could traverse the destabilizing outer membrane and incorporate into the damaged membrane. As shown in Fig. 7, R-FV-I16 and ME26 were detected in a dose-dependent manner in permeabilizing the outer membrane of *E. coli* UB1005. Both could permeabilize the outer membrane at different concentrations from 0.5 to 8 µM. At a lower concentration such as 0.5 µM R-FV-I16 has a parallel level with ME26. Compared with ME26, R-FV-I16 has a remarkable capability to permeabilize the outer membrane at a higher concentration.

**Figure 8:**
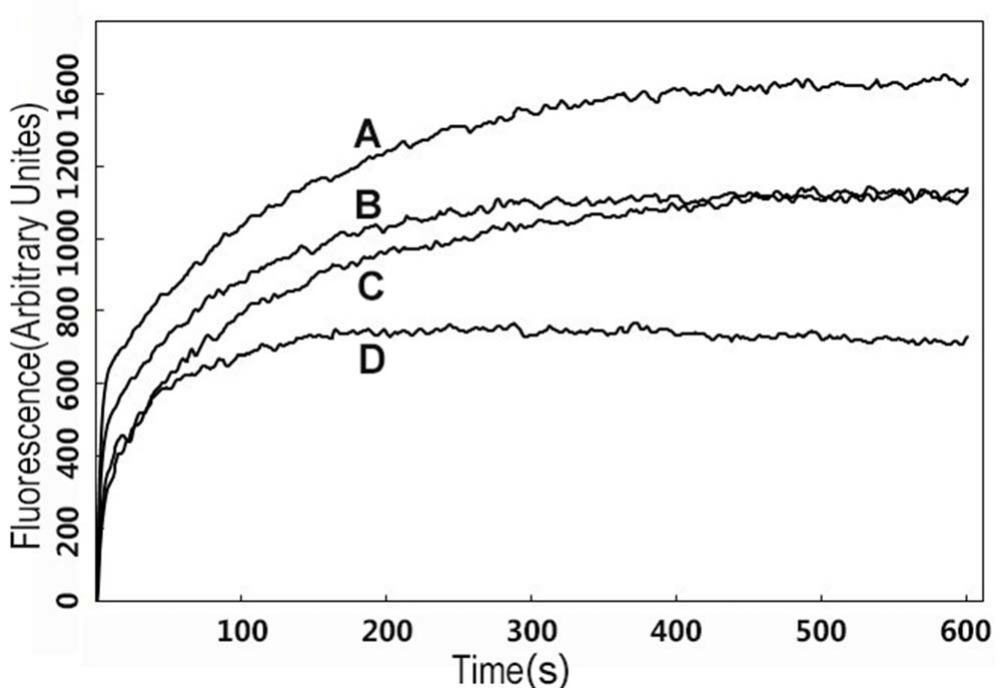
Cytoplasmic membrane variation of *E. coli* UB1005 treated by peptide and ME26. The dye (diSC_3_-5) release intensity was monitored at an excitation wavelength of 622 nm and an emission wavelength of 670 nm. The concentrations of the peptides were their 2× MIC (A, R-FV-I16; B, ME26), and 1× MIC (C, R-FV-I16; D, ME26).

Most peptides have the capability to depolarize the cytoplasmic membrane of bacteria, which could be assessed by using a membrane potential-dependent probe (diSC_3_-5) that is quenched in the cytoplasmic membrane. The chemical potential of K^+^ inside maintains a balance with outside cells by adding 0.1 M KCl to the buffer. DiSC_3_-5 would be released into medium, whereas the membrane potential is dissipated, resulting in a conspicuous increase in fluorescence. Depolarization induced by different concentrations of R-FV-I16 and ME26 was monitored over a period of 600 s, as shown in Fig. 8. The results showed that both R-FV-I16 and ME26 were able to permeabilize the bacterial cell membrane even at a lower concentration than their MICs. At the same concentration, R-FV-I16 was more effective and rapid than ME26 at permeabilizing the cytoplasmic membrane. The fluorescence intensity of R-FV-I16 (1/2 × MIC) has a in a similar manner, compared with ME26 (1 × MIC).

### Disruption to bacterial membrane integrity

**Figure 9:**
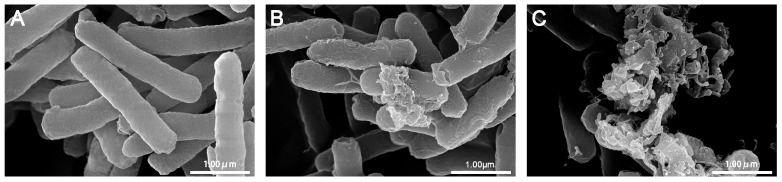
SEM micrographs of *E. coli* ATCC25922 treated by peptide at 1× MIC. (A) Control, (B) R-FV-I16 for 30 min, (C) R-FV-I16 for 120 min. The control was processed without peptide.

Numerous antibacterial peptides kill bacteria predominantly via membrane permeabilization and subsequent structural disruption. FE-SEM was used to visualize bacterial membrane damage following treatment with peptides at 1 × MIC for 30 and 120 min, respectively. As shown in Fig. 9, the membrane surface of control (without peptides) is bright and smooth. In contrast, the treatment with peptides induced significant membrane damage. After treatment with R-FV-I16 for 30 min, the membrane surface of the *E. coli* cells became rough and displayed blebbing. For a longer treatment (120 min), the membrane became absolutely rough and ruptured, and the intracellular contents had dispersed in the surface.

**Figure 10:**
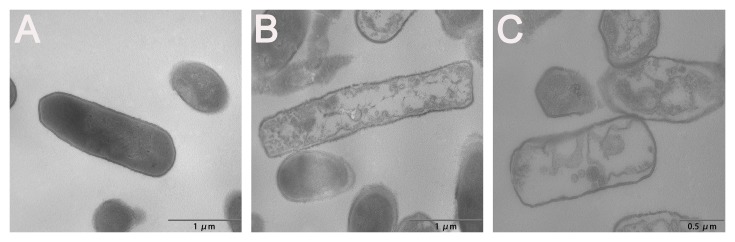
TEM micrographs of *E. coli* ATCC25922 treated by peptide at 1× MIC. (A) Control, (B) R-FV-I16 for 30 min, (C) R-FV- I16 for 120 min. The control was processed without peptide.

In addition to SEM, TEM was employed to visualize the morphology and intracellular alteration of bacteria. After 30 min of treatment with R-FV-I16, the cytoplasmic membrane of *E. coli* cells showed a slight rupture and leakage of intracellular contents. After 120 min of treatment, the significant rupture of cell membranes, the change of cellular morphology and the release of intracellular contents could be observed clearly, as shown in Fig. 10.

### Anti-biofilm activity

**Figure 11:**
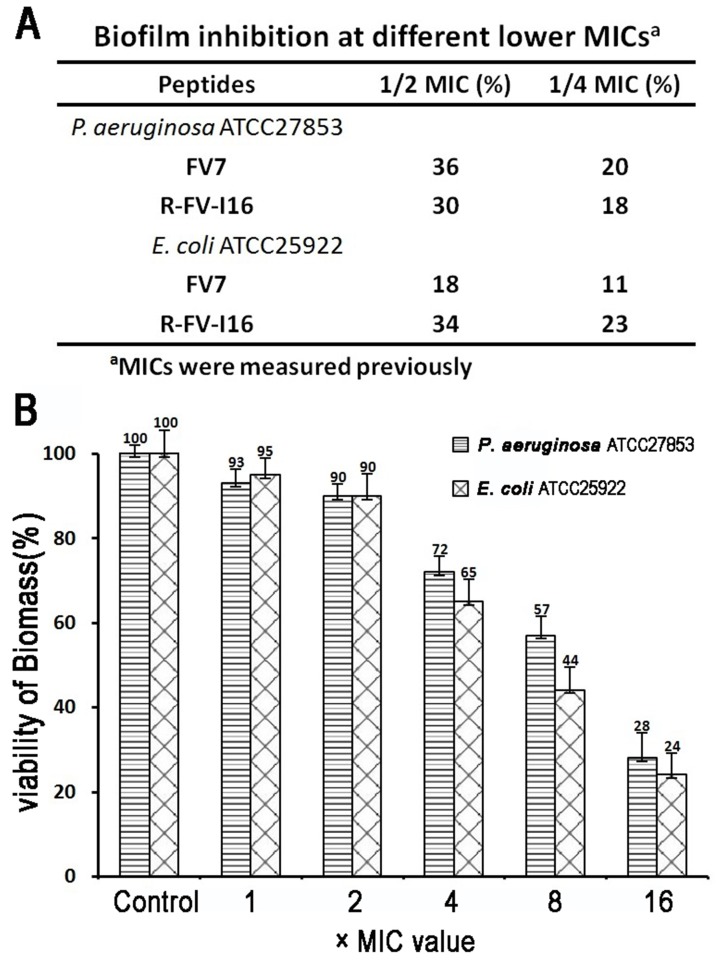
Anti-biofilm activity of the peptides. A) The capability of peptides inhibited the formation of biofilm at sub-inhibitory concentrations. B) The viability of *P. aeruginosa* and *E. coli* biofilm was reduced after 24 h treatment at higher concentrations of R-FV-I16. The tests A and B were performed at least four times in triplicate.

**Figure 12:**
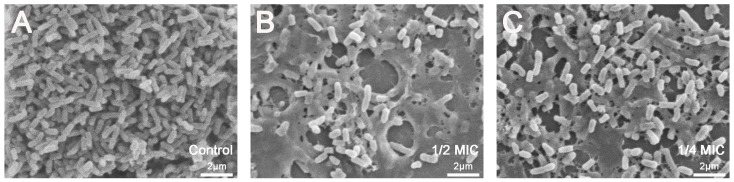
SEM micrographs of *P. aeruginosa* biofilm treated with different concentrations of R-FV-I16. A greater reduction in bacteria cell density and changer of morphology were observed at 1/2 and 1/4× MIC. (A) Control, (B) 1/2× MIC, (C) 1/4× MIC.

We investigated the anti-biofilm activity of all the peptides on *P. aeruginosa* ATCC27853 and *E. coli* ATCC25922 at sub-inhibitory concentrations and the viability assay of preformed biofilm cells at higher concentrations. The parental peptide RI16 failed to show anti-biofilm activity. From Fig. 11A, R-FV-I16 and parental peptide FV7 demonstrated a dose-dependent inhibition of biofilm formation. For the viability testing, an indirect method (MTT assay) was used to measure the decrease in the cell viability. After treating biofilms with peptides, it was observed that the amount of biomass was reduced, with increasing concentrations (1 to 16 × MIC) of R-FV-I16 (Fig. 11B). As shown in Fig. 12, the biofilm cells density decreased after treatment with R-FV-I16. The morphology of biofilm cells had a significant change.

## Discussion

The disruption of amphipathicity is an effective strategy for the design and/or optimization of α-helical AMPs [18], [19], [43]. As a general model, hybrid peptides serially combining different functional sequences such as high α-helical or β-hairpin conformation, exhibit remarkable antimicrobial activity, low hemolysis and cytotoxicity, as well as ideal cell selectively [21], [44]. Since the serial connection of different sequences indicates excessively numerous residues, one deficit existing in this design is that sharply increasing cost compromises research and clinical application. As to the embedded-hybrid pattern, a functional sequence has substituted for certain sequence, and was embedded into parental peptide, thereby the length is unchanged.

Based on the objective of this study, we designed and obtained a novel pattern retaining short sequence. At the same time, this pattern had anticipant cell selectivity and anti-biofilm activity. In this study, we disrupted the perfect amphipathicity of the α-helical peptide RI16 by replacing its central region (R6-R12) with a functional sequence (FV7) known for anti-biofilm property. Therefore, FV7 was embedded into the RI16 sequence. Parental peptide FV7 failed to show significant antimicrobial activity against planktonic cells, paradoxically, FV7 altered the thickness and morphology of biofilms, leading to a decreased number of biofilm cells of *P. aeruginosa*. That is in consistent with previous report [16]. In this study, R-FV-I16 with embedded sequence FV7 could inhibit the biofilm formation, whereas the imperfectly amphipathic α-helical structure could kill bacteria effectively. This pattern is strong evidence that hybrid peptides could combine the advantage of different domains or functional-sequences.

The results of CD spectra indicated that all the peptides displayed typical random coil structure in aqueous solution. In the anionic micellar environment (SDS), R-FV-I16 presents higher helical content than RI16. This result was possibly caused by the change of net charge [45]. R-FV-I16 had less net charge than RI16, which resulted in the higher helical content than RI16. In terms of R-FV-I16, the conformational change was induced by different environment, such as SDS and TFE. In this study, neutral SDS was used to examine the behavior of AMPs in a membrane-mimicking environment. The structure of the α-helix peptide in TFE is similar to that in SDS micelles at the acidic pH [46]. This conformational content was consistent with our hypothesis and confirmed the previous reports [24], [47]. The results were well correlated with the antimicrobial activity of these peptides.

It is important to develop AMPs as antimicrobial agents with less toxicity against host cells. The hemolytic assay showed that both parental peptides had lower hemolytic activity. Dose-response studies revealed that the hybrid peptide R-FV-I16 displayed significantly high cytotoxicity at a high concentration. Presumably, that might be because of the low hydrophobicity. Whereas it is widely known that structural parameters such as helicity and higher hydrophobicity frequently correlate with improved hemolytic activity [48], [49], which might not be the case for the peptide R-FV-I16. The peptide structure is more important than a hydrophobicity increase in determining cytotoxicity toward mammalian cells [20]. The disruption of amphipathicity by embedded a substituted sequence produced almost no hemolysis. Within an electrically neutral membrane mimetic environment (50% TFE), R-FV-I16 maintains a helical structure, it is also agreement with previous studies that as to polar substituted peptides, hemolytic activity is less correlated with self-association, hydrophobicity and amphipathicity [19]. Embedded-hybrid peptide R-FV-I16 has an inherent lower hemolytic activity, which is paralleled to both parental peptides.

In terms of MICs, the results demonstrated that R-FV-I16 possessed remarkable antimicrobial activity and broader antimicrobial spectra, compared with its parental peptides. The net positive charge, amphipathicity, and α-helix propensity are important for the antimicrobial activity. It is typically believed that there is a positive correlation between the initial electrostatic attraction of peptides and the initial electrostatic attraction of peptides, and this correlation depends on cationic charge [50], but this is not always the case. In this study, parental peptide RI16 (+12) has a higher net charge than R-FV-I16 (+8). After part net charges of RI16 were replaced by sequence FV7 (+3), and the hydrophobic tagging maintained a more rational number, the antimicrobial activity was improved significantly. It was likely that amphipathicity was an important factor to RI16, and imperfect amphipathicity could enhance cell selectivity [20]. On the other hand, hydrophobicity also likely enhances the antimicrobial activity of peptides, particularly polar and highly charged ones [51].

It is worth noting that hemolytic activity often correlates with improved antimicrobial activity because of similar determinants such as helical probability and hydrophobicity. However, this was not true in this case. Based on the MIC and MHC values, hybrid peptides appear to have ideal antimicrobial activity and maintain a low hemolytic activity.

With different salts in physiological concentration added in the medium, the antimicrobial activity of the peptide was somewhat compromised. The membrane-active mechanism illustrated that peptides could migrate toward the cell membrane by electrostatic forces between the negative charges on the outer bacteria envelope and cationic AMPs [52], [53]. The decreased MICs might be caused by weakening the electrostatic interaction between peptides and membranes in the environment of some salts, such as Na^+^ and Mg^2+^, which had a weakened effect on R-FV-I16. And this effect was particularly observed in a higher ionic strength such as in the presence of Na^+^. A previous study demonstrated that divalent ions (such as Mg^2+^) could decrease the activity of cationic AMPs [33], and it is also observed in this case. Mg^2+^ possibly interfere the electrostatic attraction and compete for membrane binding between peptides and cations, causing a reduced activity of the AMPs [52], [54]. While peptides were treated with the mixture of all the salts in physiological concentrations, the activity declined dramatically. The suppression of antimicrobial activity by proteolytic solutions and serum has hindered the clinical application. In this study, R-FV-I16 retained partial antimicrobial activity after treatment with human serum and enzymes. It is demonstrated that although some shortcomings exist, this pattern is a potential candidate for antimicrobial agents in clinical therapeutic. Biochemical stability is important for peptides used in manufacturing. Heat stability is a potential characteristic for application because many procedures such as food or feed processing involve a heating step. And the hybrid peptide R-FV-I16 had high resistance to heat.

To further investigate the interaction between peptides and membranes, membrane permeability and microscopic techniques were used. The predominant component of cell outer membranes is hydroxylated phospholipid, which keeps the negative net charge at physiological pH, such as phosphatidylglycerol (PG), cardiolipin (CL) and phosphatidylserine (PS) [55]. The peptide could insert into the cytoplasmic membrane lipid bilayer, causing the membrane permeabilization and pore/ion channel formation [56]. The outer membrane permeability assay demonstrated that as to *E. coli*, the disruption of the outer membrane occurs rapidly and this process depends on concentrations. The inner membrane permeability assay indicated that both ME26 and R-FV-I16 could permeabilize inner membrane, and R-FV-I16 had a better performance than ME26. At 1 or 1/2 × MIC concentration, the trans-membrane potential was simultaneously dissipated by the disruption of the membrane, which caused cell cytoplasmic content leakage, leading to cell death. Similar with ME26, R-FV-I16 also demonstrated a non-selective antimicrobial activity. The results of evaluation of the outer membrane permeability indicated that both peptides R-FV-I16 and ME26 could damage the outer membrane of *E. coli* UB1005, and even R-FV-I16 had a better performance than ME26. And similar performance with ME26 in cytoplasmic membrane electrical potential measurement, the result further indicated that R-FV-I16 could damage the bacterial cytoplasmic membrane, it has been widely reported that ME26 could act on cytoplasmic membranes [57], [58]. From SEM and TEM observations, R-FV-I16 had a potent interaction with membrane structure and could intensively disrupt the cell membrane and induce the leakage of the intracellular contents. Its results showed the damage of cytoplasmic membrane caused by the peptide R-FV-I16. Although the different membrane structure between Gram positive and Gram negative exists, the bacterial cytoplasmic membrane is composed of a phospholipid bilayer and proteins and encloses the contents of the bacterial cell [59]. It is common structure or component of membrane to both Gram bacteria [60]. Therefore, this kind of mechanism makes all kinds of tested bacteria seem having the same sensitivity to R-FV-I16, as above results about antimicrobial activity.

As biomaterials for clinical applications, R-FV-I16 had the potential of inhibiting biofilm formation. Compared with the parental peptide FV7, R-FV-I16 exhibited reasonable anti-biofilm activity. Especially as to *E. coli* biofilms, R-FV-I16 had a better performance than FV7. The capability of FV7 to inhibit biofilm formation was associated with a decreased number of bacterial cells reaching the surface [16]. With potent antibacterial activity, R-FV-I16 could kill the preformed biofilm cells at high concentration. As an embedded-hybrid pattern, R-FV-I16 has anti-biofilm activity in applications.

## Conclusions

In this study, we have provided a new pattern for designing hybrid peptides by embedding functional sequence into other peptide. This approach broadens their antibacterial activity and maintains their anti-biofilm activity. The excellent cell selectivity was due to the disruption of the amphipathicity of α-helical AMPs, and the anti-biofilm activity is derived from the parental peptide. The peptide R-FV-I16 could damage the membrane and kill the cell by permeabilizing the outer and inner membranes of bacterial cells, depolarizing cytoplasmic membrane. Although the peptide in our study has some drawbacks, the rational design will be useful for future assessments of peptide models. With ongoing optimization, embedded-hybrid antimicrobial peptide will be useful as new antimicrobial agents against Gram-negative and Gram-positive bacteria.
